# Validation of a case definition to define chronic dialysis using outpatient administrative data

**DOI:** 10.1186/1471-2288-11-25

**Published:** 2011-03-01

**Authors:** Fiona M Clement, Matthew T James, Rick Chin, Scott W Klarenbach, Braden J Manns, Robert R Quinn, Pietro Ravani, Marcello Tonelli, Brenda R Hemmelgarn

**Affiliations:** 1 Department of Medicine, University of Calgary, 2500 University Dr. NW, Calgary, Alberta, T2N 1N4, Canada; 2 Department of Community Health Sciences, University of Calgary, 2500 University Dr. NW, Calgary, Alberta, T2N 1N4, Canada; 3 Libin Cardiovascular Institute, University of Calgary, 2500 University Dr. NW, Calgary, Alberta, T2N 1N4, Canada; 4 Calgary Population and Public Health Institute, University of Calgary, 2500 University Dr. NW, Calgary, Alberta, T2N 1N4, Canada; 5 Department of Medicine, University of Alberta, 2J2.00 WC Mackenzie Health Sciences Centre, Edmonton, Alberta, T6G 2R7, Canada

## Abstract

**Background:**

Administrative health care databases offer an efficient and accessible, though as-yet unvalidated, approach to studying outcomes of patients with chronic kidney disease and end-stage renal disease (ESRD). The objective of this study is to determine the validity of outpatient physician billing derived algorithms for defining chronic dialysis compared to a reference standard ESRD registry.

**Methods:**

A cohort of incident dialysis patients (Jan. 1 - Dec. 31, 2008) and prevalent chronic dialysis patients (Jan 1, 2008) was selected from a geographically inclusive ESRD registry and administrative database. Four administrative data definitions were considered: at least 1 outpatient claim, at least 2 outpatient claims, at least 2 outpatient claims at least 90 days apart, and continuous outpatient claims at least 90 days apart with no gap in claims greater than 21 days. Measures of agreement of the four administrative data definitions were compared to a reference standard (ESRD registry). Basic patient characteristics are compared between all 5 patient groups.

**Results:**

1,118,097 individuals formed the overall population and 2,227 chronic dialysis patients were included in the ESRD registry. The three definitions requiring at least 2 outpatient claims resulted in kappa statistics between 0.60-0.80 indicating "substantial" agreement. "At least 1 outpatient claim" resulted in "excellent" agreement with a kappa statistic of 0.81.

**Conclusions:**

Of the four definitions, the simplest (at least 1 outpatient claim) performed comparatively to other definitions. The limitations of this work are the billing codes used are developed in Canada, however, other countries use similar billing practices and thus the codes could easily be mapped to other systems. Our reference standard ESRD registry may not capture all dialysis patients resulting in some misclassification. The registry is linked to on-going care so this is likely to be minimal. The definition utilized will vary with the research objective.

## Background

The global prevalence of end-stage renal disease (ESRD) requiring treatment with dialysis or kidney transplantation continues to increase [1,2]. Patients with ESRD experience far greater morbidity, mortality and health care costs than members of the general population, and studies evaluating health outcomes in this high-risk population are required worldwide [1,2].

Administrative health care databases offer an efficient and accessible approach to studying outcomes in large populations[3]. Physician billing claims data are one data source for identifying cases of ESRD because they are routinely collected for physician reimbursement, often span wide geographic areas, and have the potential to capture both in-hospital and outpatient encounters within a healthcare system[4]. However, before such data sources can be widely adopted for use in research where identification of cases of ESRD is critical, the validity of algorithms used to define case definitions of ESRD requires evaluation.

Limited data demonstrate the validity of administrative data algorithms for identifying patients requiring chronic hemodialysis or peritoneal dialysis. Prior studies have assessed acute kidney injury [5-7], as well as the validity of using *inpatient *administrative data to identify chronic dialysis patients [8-13]. The two previous studies considering chronic dialysis in the outpatient setting have considered diagnostic codes [14,15], not procedural codes as are considered in this study. This is of particular importance as the majority of contemporary ESRD patients receive chronic dialysis as outpatients. We therefore did this study to determine the validity of algorithms derived from outpatient physician billing claims for defining chronic dialysis, compared to the reference standard of an ESRD registry.

## Methods

### Study Population

A cohort was identified from the Alberta Kidney Disease Network (AKDN - http://www.akdn.info) laboratory database to form the study population. The AKDN is a prospective data collection initiative of routine laboratory tests on all patients in the province of Alberta (population approx. 3 million) Canada, resulting in a population-based geographically inclusive database [16]. Patients identified from laboratory data are followed prospectively with linkage to administrative and other computerized sources to obtain detailed information including socio-demographic data, clinical data including comorbidities, health care encounters, health care costs, death, and kidney-related outcomes. The study cohort included patients aged 18 and older who had at least 1 outpatient serum creatinine between Jan 1 2008 and Dec 31 2008. Although a general population cohort would be optimal, our selected study population introduces minimal, if any, bias as anyone "at-risk" of ESRD or evaluated for or receiving chronic dialysis was expected to have received serum creatinine measurement as part of their routine clinical assessment.

### Data sources

Patients treated for ESRD in Alberta are cared for by the Northern Alberta (NARP) and Southern Alberta (SARP) Renal Programs [17]. These programs are responsible for providing ESRD care including chronic dialysis within their geographic area. Each program maintains a prospective patient registry of all chronic dialysis patients, and captures detailed demographic and clinic data, including date of initiation of dialysis. Patients are enrolled at the time of first dialysis for ESRD (first hemodialysis session or first flushing of peritoneal dialysis catheter), or, for patients who initiate dialysis for acute kidney injury, when the attending nephrologist deems that dialysis will be chronic. The NARP and SARP registries were used to identify prevalent and incident dialysis patients from January 1, 2008 to December 31, 2008 (considered the reference standard). Prevalent cases were first identified on Jan 1 1999, with additional incident dialysis patients identified from that date forward. Non- Alberta residents were excluded.

Physicians in Alberta submit claims for reimbursement of services to Alberta Health and Wellness, the provincial health ministry, (the universal health care provider for the province of Alberta); claims are stored in a database which contains information on patients' personal health number, physician unique identifier, up to 3 ICD-9 diagnosis codes and 1 procedure code. Procedure codes are captured using the Canadian Classification of Diagnostic, Therapeutic and Surgical Procedures (CCP, which was developed by Statistics Canada to accompany the International Classification of Diseases version 9 (ICD-9) [18]. Physician claims capture all of the outpatient physician services and the majority of the inpatient services. All chronic dialysis patients in the province of Alberta are cared for by nephrologists, who are compensated either using a fee-for-service or salaried model. Regardless of compensation method, physicians are required to submit claims for all patient encounters.

### Defining Chronic Dialysis using Administrative Data

We identified all patients with outpatient dialysis physician claims (Table 1) occurring from Jan 1 2008 to Dec 31 2008. We evaluated 4 different case definitions for chronic dialysis patients based on varying the number and timing of physicians claims for dialysis: 1) At least 1 outpatient claim, 2) At least 2 outpatient claims, 3) At least 2 outpatient claims at least 90 days apart and 4) Continuous outpatient claims at least 90 days apart with no gap in claims greater than 21 days. We evaluated algorithms employing a 90 day period of claims to be congruent with other current administrative data definitions developed using inpatient data [19,20].

**Table 1:** Administrative billing codes used to define outpatient chronic dialysis

Canadian Classification of Diagnostic, Therapeutic and Surgical Procedures Codes	Code Description
13.99A	Hemodialysis treatment, unstable patient
13.99B	Hemodialysis treatment, stable patient
13.99C	Assessment and management of an unstable patient with acute/chronic renal failure treated by peritoneal dialysis
13.99D	Assessment and management of a stable patient with chronic renal failure treated by peritoneal dialysis
13.99O	Management of dialysis patients on home dialysis or receiving treatment in a remote hemodialysis unit (per week)
13.99OA	Management of patient on hemodialysis or peritoneal dialysis (per week)

### Comorbidities and other outcomes

Demographic data were determined from the provincial administrative data files. Diabetes mellitus and hypertension were identified from hospital discharge records and physician claims based on validated algorithms [4,21]. The Charlson comorbidities were calculated using the validated algorithms applied to physician claims and hospitalization data [22,23]. Any comorbidity identified during the 3 year period prior to cohort entry was included. To ascertain death, patients were followed up from their start date of dialysis, defined either by the first recorded date in the registry or the date of the second of the outpatient claims when the administrative data definition was used, until March 31, 2009 to ensure a minimum of 90 days of follow-up for all patients. Patients who met the case definition and subsequently died or moved out of the province (lost to follow-up) were included in analyses

### Statistical Analysis

Basic descriptive statistics were used to describe demographic features and comorbidities for the overall cohort, the NARP/SARP dialysis cohort. Table 2 outlines the analytic framework adopted. We subsequently calculated positive agreement, sensitivity, positive predictive value (PPV), for each case definition, using the NARP/SARP registry data as the reference standard [24]. Positive agreement is the conditional probability, given the reference standard is positive, the administrative data definition is also positive [25]. Thus, the positive agreement will explore if there is an imbalance between the likelihood of agreeing on positive and negative cases. The kappa-statistic was used to assess overall agreement between the registry and the billing data. Landis and Koch categorize Kappa into five categories: less than 0.2 indicating "poor agreement", 0.21 to 0.40 indicating "fair agreement", 0.41 to 0.60 indicating "moderate agreement, 0.61 to 0.80 indicating "substantial agreement" and greater than 0.81 indicating "near perfect agreement"[26]. We did not report specificity, negative agreement, or negative predictive value (NPV) as the large size of the non-diseased population (n = 1.11 million) and low incidence of ESRD in the general population makes these measures insensitive to changes in the case definitions. SAS version 9.2 was used for all analyses. Ethics approval was obtained from the Conjoint Health Research Ethics Board at the University of Calgary.

**Table 2:** Reported measures of agreement: analytic framework

		Registry definition (Reference Standard)		
		
		+	-	Total
**Administrative record**	+	a	b	a+b
	
	-	c	d	c+d

	Total	a+c	b+d	N

Abbreviations: a: true positives; b: false positives; c: false negatives; d: true negatives; N: total validation sample

**Positive agreement **= 2a/[2a+b+c]

The measure of agreement in positive cases; the number of true positives divided by all of the positives defined by both data sources. Positive agreement will show imbalance in the agreement between positive and negative responses.

**Sensitivity **= a/(a+c)

The proportion of those on dialysis according to the administrative data among those positive cases identified in reference standard. The ability of administrative data to correctly identify individuals on dialysis according to the reference standard

**Positive Predictive Value (PPV) **= a/(a+b)

The proportion of those on dialysis according to the reference standard among those positive cases identified in the administrative data

**Kappa **= [(a+d)/N - ((a+b)(a+c) + (c+d)(b+d))/N^2^]/[1-((a+b)(a+c)+(c+d)(b+d))/N^2^]

The agreement between the administrative data and reference standard above what is expected by chance

## Results

In total 1,118,097 individuals had at least 1 out-patient serum creatinine measure from Jan 1 2008 to Dec 31 2008. During that period 2,227 chronic dialysis patients (0.20% of the total study population) were registered in the ESRD registry. Table 3 presents the baseline characteristics of the overall population, the reference standard dialysis cohort and the cohort resulting from each of the administrative data definitions. The characteristics of the overall cohort are similar to the general Alberta population [27]. As expected, the dialysis cohort was older (64.0 vs. 52.6 y), had a higher prevalence of diabetes (54.5% vs. 12.7%), hypertension (89.0% vs. 34.7%) and a higher burden of comorbid disease (median number of Charlson comorbidities 3 vs. 0) compared to the total population. As the administrative data definition became more restrictive, the cohort became slightly older with a moderately higher burden of diabetes and hypertension.

**Table 3:** Baseline cohort characteristics

			Administrative data definitions			
	**Total Population** **(n = 1,118,097)**	**Reference Standard** **(n = 2,227)**	**1 outpt claim** **(n = 2324)**	**At least 2 outpt claims** **(n = 2171)**	**At least 2 outpt claims 90 days apart** **(n = 1657)**	**Continuous claims** **(n = 1508)**

Mean Age (SD)	52.6 (17.63)	64.0 (15.62)	62.8 (16.2)	63.2 (16.1)	63.4 (16.2)	64.0 (16.0)
Male (%)	43.2	60.3	59.5	60.1	59.0	59.4
Diabetes (%)	12.7	54.5	51.9	52.7	53.7	54.2
Hypertension (%)	34.7	89.0	88.5	89.5	91.9	92.1
Median number of Charlson Comorbidites (median, IQR*)	0 (0,1)	3 (2,5)	3 (2,5)	3 (2,5)	3 (2,5)	3 (2,5)
Median number of Outpt Physician Claims in 2008 (median, IQR*)	8 (4,14)	67 (24,136)	80 (35,142)	90 (42,145)	124 (69,155)	129 (81,160)

*Interquartile range

The chronic dialysis case definitions based on 1 outpatient claim and 2 outpatient claims resulted in similar prevalence estimates to the reference standard (0.21% and 0.19% respectively). The other two definitions, incorporating claims spanning 90 days, underestimated the prevalence (Table 4). The positive agreement was highest when the definition using 2 outpatient claims was considered. The four coding algorithms for dialysis resulted in sensitivities ranging from 0.58 (Continuous outpatient claims) to 0.81 (at least 1 outpatient claim). The PPVs ranged from 0.77 (at least 1 outpatient claim) to 0.86 (Continuous outpatient claims). The three definitions requiring at least 2 outpatient claims resulted in kappa statistics between 0.60-0.80 indicating "substantial" agreement [26]. "At least 1 outpatient claim" resulted in "excellent" agreement with a kappa statistic of 0.81, however, given the size of the true negative population this must be interpreted with caution [24].

**Table 4:** Validity of physician billing chronic dialysis case definitions compared with reference standard registry case definition

Definitions	Admin (+) n (prev.) (a+b)	Registry (+) Admin (+) n (a)	Registry (-) Admin (+) n (b)	Registry (+) Admin (-) n (c)	Registry (-) Admin (-) n (d)	Positive agreement	Sensitivity	PPV	Kappa
1 outpatient claim	2324 (0.21%)	1805	519	422	1,115,351	0.793	0.811	0.777	0.793

2 outpatient claims	2171 (0.19%)	1751	420	476	1,115,450	0.796	0.786	0.807	0.796

2 outpatient claims at least 90 days apart	1657 (0.15%)	1406	251	821	1,115,619	0.724	0.631	0.848	0.724

Continuous outpatient claims for at least 90 days with no gaps greater than 21 days	1508 (0.13%)	1295	213	932	1,115,657	0.693	0.582	0.859	0.693

Abbreviations: prev - Prevalence = Admin (+)/total N

## Discussion

All four physician claims-based case definitions assessed resulted in "substantial" agreement with our reference standard registry definition for chronic dialysis. One outpatient claim for dialysis was the most sensitive definition, while more complicated definitions exhibited modest increases in positive predictive value. The optimal administrative data definition may vary with the research objective. For example, when seeking to maximize identification of dialysis as an outcome an approach based on at least 1 outpatient claim may be preferable. In contrast, when establishing a cohort of patients with ESRD receiving chronic dialysis that includes the fewest non-diseased cases being captured, the use of continuous outpatient claims may be better suited.

Some of the discrepancies between our registry and physician claims algorithms for chronic dialysis likely relate to differences in the classification of patients who receive temporary dialysis or who die soon after initiating dialysis Traditionally, administrative algorithms and national registries, such as the USRDS, have required a 90-day timeframe to define chronic dialysis [19,20]. Although this approach avoids identification of patients who receive temporary dialysis then recover renal function within 3 months, it introduces survivor bias and does not capture chronic dialysis patients that may begin dialysis but die before meeting the inclusion criteria of the definition. Our study demonstrates that approaches based on 1 or 2 outpatient dialysis claims are substantially more sensitive than definitions based on 90 days of claims, although this definition may include some patients who would not be classified as receiving chronic dialysis in a registry (false positive cases). Utilizing a definition that does not require the patient to survive a certain amount of time eliminates any potential survival bias and allows studies of the patient group that begin dialysis and die soon after. However the limitation of this definition is that it may also include patients with acute kidney injury requiring dialysis for a short period who subsequently recover their renal function and no longer require dialysis. Furthermore, estimates of disease incidence and outcomes will not be comparable to studies based on most existing national registries.

Establishing the validity of an outpatient administrative data definition for chronic dialysis will allow researchers to utilize physician billing claims data to assess outcomes and form cohorts. This is of international relevance, even in countries where established dialysis registries are available. In the United States, not all researchers have the means to access the USRDS. In other registries from other countries often only cross-sectional, regional data with limited outcomes are available. Thus, validated methods for identifying chronic dialysis patients using billing claims data would be useful for in health services research.

We found that the use of physician claims data resulted in the classification of patients as receiving dialysis who were not identified as such in our registry (false positives). Most of these patients were removed from the case definition when algorithms which required claims to span 90 days were used. This is in-keeping with the hypothesis that these events may be acute kidney injury cases or patients who were initiated on dialysis but subsequently recovered renal function; i.e., those not considered chronic dialysis patients and thus not captured in the registry. We also found that physician claims failed to identify some patients captured in the registry (false negatives). As Alberta Health and Wellness does not employ any formal quality assurance or correction process, this may be due to missed billings, billing errors, billings made by physicians on alternative payment plans (shadow billing) or miscoding present in administrative data sources, as the number of such patients decreased when algorithms that required less intensive physician claims were employed.

To our knowledge, this is the first study to look at using outpatient administrative data sources using procedure codes to define chronic dialysis. Others have developed algorithms for acute kidney injury and chronic kidney disease using inpatient administrative data [5-13]. Given that the majority of chronic dialysis patients are treated in the outpatient setting, administrative data algorithms limited to inpatient encounters are likely to perform poorly when compared against a reference standard. Three previous studies have included outpatient claim data [14,15,28]. However, Kern et al. excluded chronic dialysis patients, focusing on the validity of administrative data to define chronic kidney disease defined by eGFR <60 ml/min/1.73 m^2 ^[28]. Neither Weintraub et al. nor Wilchesky et al. included procedural codes [14,15]. Their work was limited to ICD-9-CM diagnosis codes for chronic renal failure. Thus, our study is novel, and could facilitate further health services research in a high risk population with ESRD who experience very high morbidity, mortality, and health care costs.

Our study does have several limitations. First, the billing codes used are from the Canadian Classification of Diagnostic, Therapeutic and Surgical Procedures (CCP); a classification system developed and applied in Canada. However, most countries have similar billing practices and billing codes that could be mapped to the CCP codes. Second, we used a provincial registry of all chronic dialysis patients as the reference standard. Although this registry is geographically inclusive, some dialysis patients may be omitted from the registry in error, thereby resulting in misclassification. However, as this registry is linked to ongoing dialysis treatment, the number of patients not registered is expected to be small. Third, our study did not distinguish between dialysis modalities (hemodialysis versus peritoneal dialysis, or in-centre versus home dialysis), and the accuracy of patient registry and physician claims in these settings may vary. However, prior research has reported limitations in the accuracy of administrative data for identifying the timing of changes between dialysis modalities suggesting that administrative data sources may be better suited to the general identification of patients receiving chronic dialysis rather than a specific modality [29].

## Conclusions

We found that outpatient physician claims identified patients receiving chronic dialysis with "substantial" agreement to a reference standard dialysis registry definition. The use of 1 or 2 outpatient claims was most sensitive; however, had modestly lower positive predictive value than claims spanning 90 days or continuous claims. Given the variation in the way clinicians, researchers, and research tools define chronic dialysis, the optimal physician claims based definition will vary with the research objective.

## Competing interests

The authors declare that they have no competing interests.

## Authors' contributions

All the authors contributed to the conception and design. FC, MJ, RC and BR contributed to the data analysis and drafted the report. All of the authors contributed to the interpretation of data, critically revised the manuscript for important intellectual content and approved the final version submitted for publication.

## Pre-publication history

The pre-publication history for this paper can be accessed here:

http://www.biomedcentral.com/1471-2288/11/25/prepub
